# Interplay between CCN1 and Wnt5a in endothelial cells and pericytes determines the angiogenic outcome in a model of ischemic retinopathy

**DOI:** 10.1038/s41598-017-01585-8

**Published:** 2017-05-03

**Authors:** Sangmi Lee, Menna Elaskandrany, Lester F. Lau, Douglas Lazzaro, Maria B. Grant, Brahim Chaqour

**Affiliations:** 1 Department of Cell Biology, State University of New York (SUNY), Downstate Medical Center, College of Medicine, Brooklyn, NY 11203 USA; 2 Department of Biochemistry and Molecular Genetics, University of Illinois at Chicago College of Medicine, Chicago, IL 60607 USA; 3 Departments of Ophthalmology, Indiana University School of Medicine, Indianapolis, IN 46202 USA; 4 Department of Ophthalmology, Downstate Medical Center, Brooklyn, NY 11203 USA

## Abstract

CYR61-CTGF-NOV (CCN)1 is a dynamically expressed extracellular matrix (ECM) protein with critical functions in cardiovascular development and tissue repair. Angiogenic endothelial cells (ECs) are a major cellular source of CCN1 which, once secreted, associates with the ECM and the cell surface and tightly controls the bidirectional flow of information between cells and the surrounding matrix. Endothelium-specific CCN1 deletion in mice using a cre/lox strategy induces EC hyperplasia and causes blood vessels to coalesce into large flat hyperplastic sinuses with no distinctive hierarchical organization. This is consistent with the role of CCN1 as a negative feedback regulator of vascular endothelial growth factor (VEGF) receptor activation. In the mouse model of oxygen-induced retinopathy (OIR), pericytes become the predominant CCN1 producing cells. Pericyte-specific deletion of CCN1 significantly decreases pathological retinal neovascularization following OIR. CCN1 induces the expression of the non-canonical Wnt5a in pericyte but not in EC cultures. In turn, exogenous Wnt5a inhibits CCN1 gene expression, induces EC proliferation and increases hypersprouting. Concordantly, treatment of mice with TNP470, a non-canonical Wnt5a inhibitor, reestablishes endothelial expression of CCN1 and significantly decreases pathological neovascular growth in OIR. Our data highlight the significance of CCN1-EC and CCN1-pericyte communication signals in driving physiological and pathological angiogenesis.

## Introduction

The expansion and/or regeneration of functional blood vessels occur by sprouting angiogenesis, which involves a series of key events including formation of new branches by migration and proliferation of endothelial cells (ECs), fusion of sprouts to form circuits and maturation and stabilization of the vascular network through coverage with mural cells such as pericytes^1^. This process is accompanied by the simultaneous deposition of a basement membrane (BM), remodeling of blood vessels into morphologically recognizable arteries, capillaries, and veins and pruning of unwanted and dysfunctional branches. Pathological angiogenesis is viewed as an overshoot reaction of this process leading to the formation of more than necessary, yet leaky, blood vessels that compromise the proper function and survival of the tissue. In particular, aberrant interactions between ECs and mural cells as seen in numerous genetic mouse models result in severe and often lethal vascular defects^2^. Abnormal interactions between the 2 cell types have been implicated in a number of human pathological conditions, including proliferative retinopathy, tumor angiogenesis, ectopic tissue calcification and CADASIL, a human stroke and dementia syndrome affecting the same type of vessels^3^.

Cell-cell and cell-matrix communication is critical for the angiogenic process to proceed properly both during normal development and regeneration^4^. Studies have suggested that the bidirectional signals between ECs and pericytes mark the end of vessel plasticity and reflect the quiescent state of newly formed vascular networks^5^. The addition of pericytes to cocultures of ECs and astrocytes stabilized capillary-like structures *in vitro* ^6^ while pericyte-deficient microvessels in genetically modified mice became hyperplastic and/or permissive for angiogenic sprouting^7^. Pericytes are typically embedded in a BM that is seamlessly merged with that of ECs. This shared extracellular matrix (ECM) structure stretches over the pericyte–endothelial interface and contains gaps/interruptions of 2 kinds: (1) adhesion plaques which anchor pericytes directly to ECs via ECM-integrin interactions to support the transmission of contractile forces from one cell type to another; and (2) peg-and-sockets structures that allow direct exchange of molecular signals between ECs and pericytes. Cytokines and growth factors such as platelet-derived growth factor-B, transforming growth factor (TGF)-β and angiopoietin 1 have been proposed to mediate EC-pericyte interactions during physiological angiogenesis^8–10^. However, the effector molecules involved in pathological intercellular signaling at the cell-matrix and EC-pericyte interaction sites are unknown.

Candidate proteins capable of regulating the bidirectional flow of information between ECs and pericytes in vascular diseases include the CCN1 protein of the CCN (CYR61/CTGF/NOV) family^11, 12^. CCN1 is an immediate-early gene-encoded ECM protein produced by mesenchymally- and ectodermally-derived cells at sites of inflammation and active angiogenesis^13, 14^. The 40-kDa CCN1 protein functions primarily through direct binding to distinct integrin receptors (e.g., α_v_β_3_, α_M_β_2_, α_6_β_1_) and triggers signal transduction events that culminate into cell adhesion, migration, proliferation, gene expression, differentiation, survival and even apoptosis. CCN1-integrin receptor interactions are cell type- and context-dependent and result in the activation of signaling cascades involving mitogen-activated protein kinase, NF-κB, tyrosine kinases, and/or Akt/PKB with subsequent initiation of transcription of gene targets^15^. In addition, CCN1 modulates the activities of several growth factors and cytokines, including tumor necrosis factor (TNF)-α, VEGF, Wnt and matrix-metalloproteinases (MMPs), and may thereby act as an integrator of multiple signals affecting a broad array of biological processes.

CCN1 is increasingly being recognized as important in developmental angiogenesis and in pathological processes such as fibrosis and oncogenesis^11, 16^. *In vitro* studies have shown that CCN1 promotes directed migration of ECs and potentiates the release of angiogenic factors from the ECM which may contributes to the overall process of neovascularization *in vivo* ^17^. Our group has studied the role of CCN1 signals in sprouting angiogenesis which is primarily driven by VEGF^18^. We found that CCN1 activity was integrated with VEGF receptor 2 (VEGF-R2) activation and downstream signaling pathways required for tubular network formation. In ECs, CCN1-integrin binding regulates the expression of, and association between, Src homology 2 domain-containing protein tyrosine phosphatase-1 and VEGF-R2, which leads to rapid dephosphorylation of specific VEGF-R2 tyrosine residues and attenuation of VEGF-induced EC hyperproliferation. In addition, CCN1 determines Notch-dependent vessel specification by regulating the expression of Delta like canonical Notch ligand 4. However, despite these advances in our understanding of CCN1 function in vascular biology, the range of CCN1 actions and potential implications in the pathogenesis of ischemic diseases remain unknown. Here we investigate how CCN1 expression, or lack thereof, affects bidirectional signaling between ECs and pericytes and defines the angiogenic outcome under pathological conditions.

## Results

### Retinal vascular expression of CCN1

**Figure 1:**
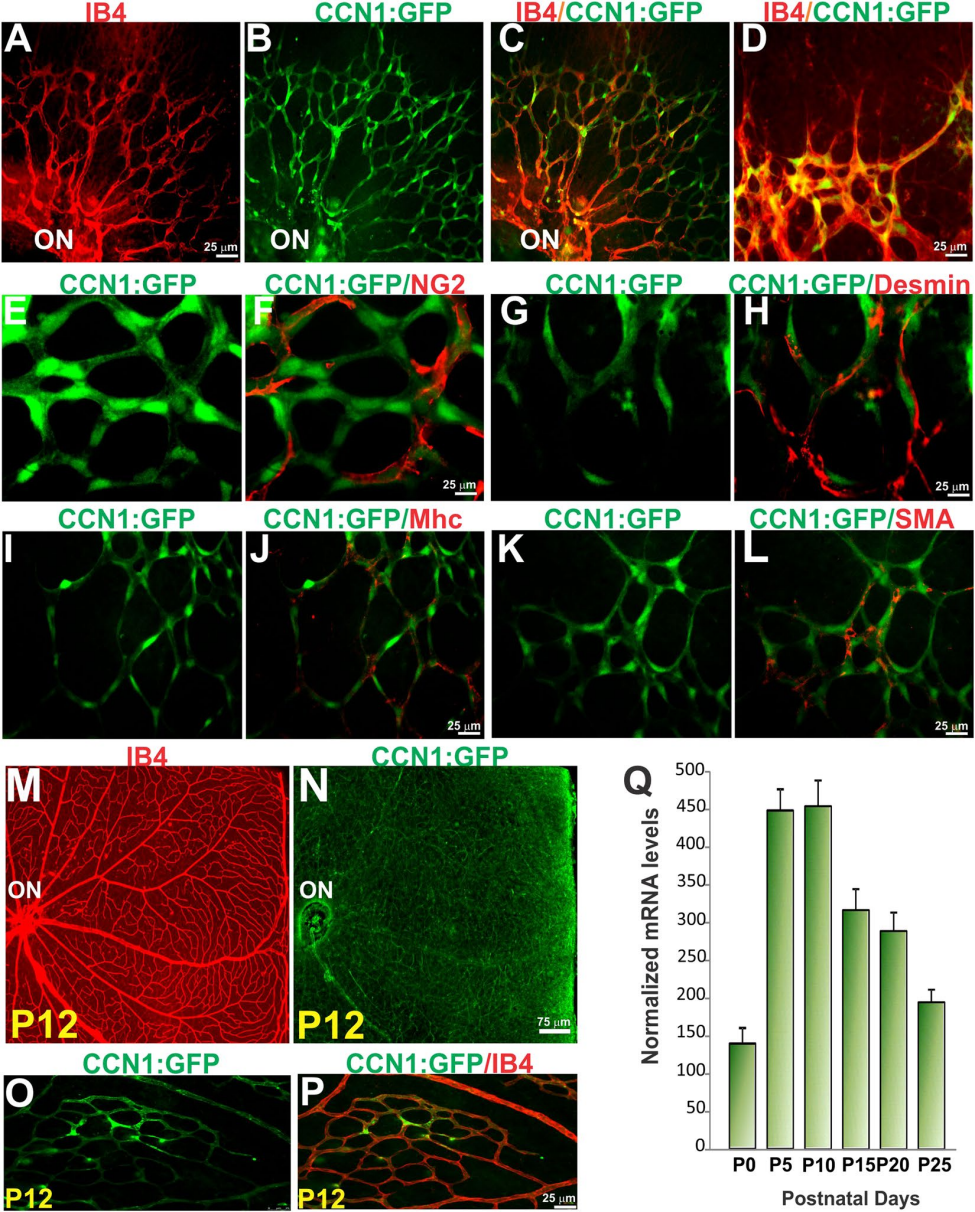
Expression of CCN1 during postnatal vascular development in the retina. (**A–D**) Flat-mounted preparations of retinas from transgenic mice expressing the GFP reporter gene under the control of the CCN1 promoter were analyzed for cellular localization of the CCN1:GFP signal in the growing vasculature. Images shown depict colocalization in P4 retinas of the CCN1:GFP signal with the endothelium-specific marker IB4. (**E–P**) The CCN1:GFP signal does not colocalize with mural cell markers including NG2, desmin, myosin heavy chain (mhc) and smooth muscle α-actin (SMA). Retinas with completely formed vasculature at P16 expressed residual amount of the CCN1:GFP in the vascular wall (ON: optic nerve). (**Q**) The transcript levels of the endogenous CCN1 gene during postnatal development of the retinal vasculature was determined by qPCR and normalized to 18S ribosomal RNA. (n = 5).

To determine the tissue distribution and cellular sources of CCN1 during angiogenesis, we analyzed flat-mounted preparations of postnatal murine retinas from a CCN1 promoter: green fluorescent protein (GFP) mouse line (MGI: 4846966) which carries the GFP reporter gene downstream of a large CCN1 promoter segment^19^. As shown in Fig. 1A–D, the CCN1:GFP signal was intense in vascular ECs marked by isolectin B4 (IB4) particularly in the sprouting vascular front of the superficial capillary plexus. Mural cells of the capillaries and precapillary arterioles and venules consist largely of pericytes which are heterogeneous in both their morphology, orientation and the markers they express. However, CCN1:GFP signal did not colocalize with neuron-glial 2 (NG2)-positive pericytes in the vascular wall (Fig. 1E,F). Similarly, CCN1:GFP signal was not present in mural cells marked by αSMA, myosin heavy chain (Mhc) and desmin labeling (Fig. 1G–L). When the retinal capillary plexuses were completely formed, the CCN1:GFP signal was attenuated but remained residually expressed in ECs (Fig. 1M–P). Quantitative analyses showed a progressive increase of the CCN1 mRNA levels during the early stages of retinal angiogenesis with a peak at P10 followed by a rapid decrease of CCN1 transcript levels as the mature capillary plexuses were completely formed (Fig. 1Q). This expression pattern is consistent with a role of CCN1 in the regulation of the phenotypic plasticity of ECs during sprouting angiogenesis.

### Pericytes as a cellular source of CCN1 under ischemic conditions

**Figure 2:**
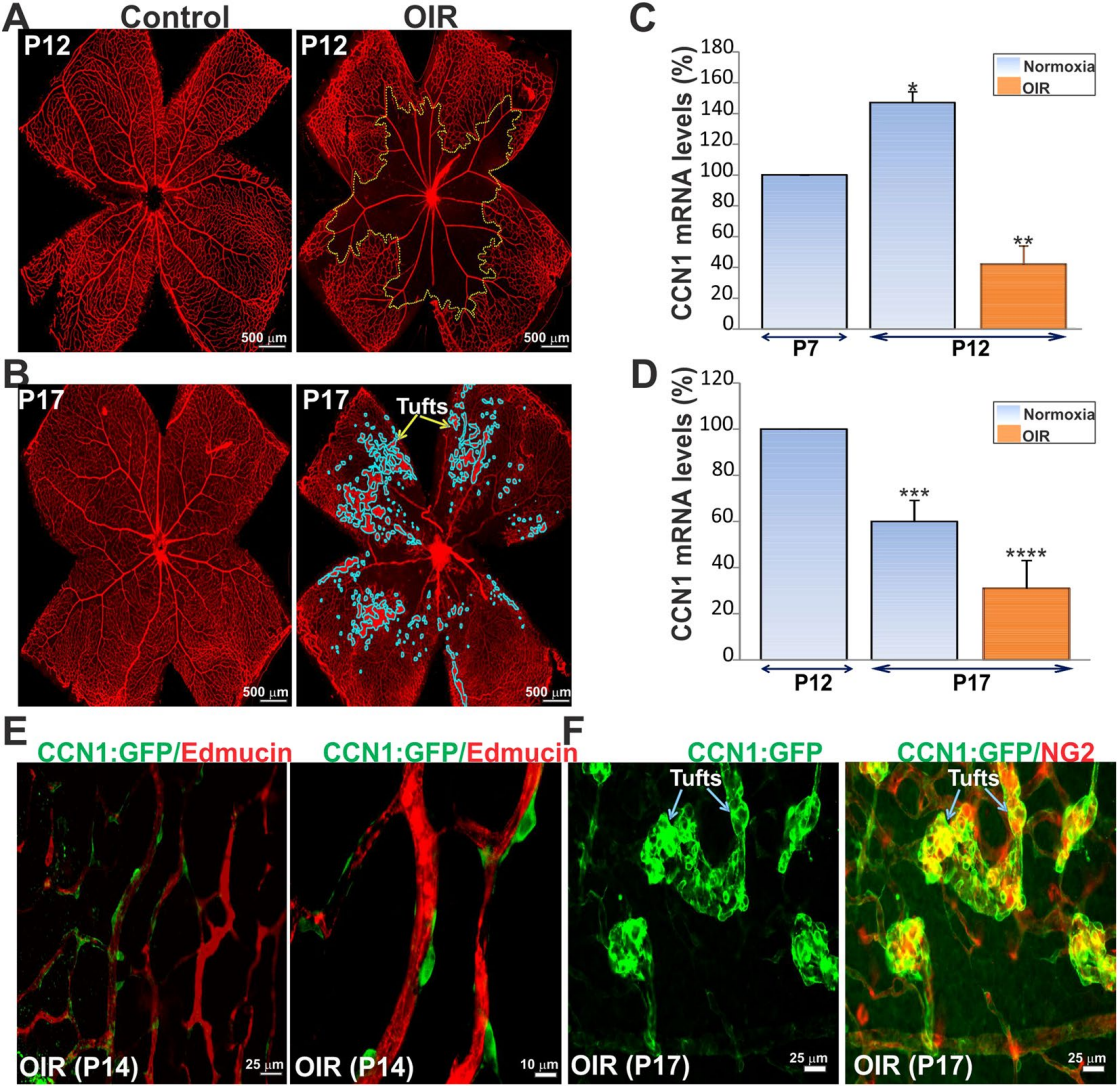
OIR-induced pre-retinal neovasculatization is associated with the expression of CCN1 in pericytes. (**A**,**B**) Representative flat mount preparations of IB4-stained retinas from control and OIR mice. Mouse pups were placed at P7 under hyperoxia (75% oxygen) or ambient air (normoxia) for 5 days. Mice were returned to ambient air until P17. Areas of vaso-obliteration and preretinal neovascular tufts as determined by computer-assisted image analyses are shown with dotted yellow lines and blue outline respectively. (**C**,**D**) Steady state mRNA levels of CCN1 following the hyperoxic (P7 to P12) and ischemic (P12 to P17) phases of OIR as determined by qPCR. CCN1 mRNA levels were normalized to those of 18S rRNA. (*n* = 4). *p < 0.05 *versus* P7 (normoxia); **p < 0.001 *versus* P12 (normoxia); ***p < 0.05 *versus* P12 (normoxia); ****p < 0.05 *versus* P17 (normoxia). (**E**,**F**) Flat mounted preparations of retinas of the CCN1:GFP reporter mice subjected to OIR. Retinas were collected during the posthyperoxic phase at either P14 or P17 and stained with the endothelial marker endomucin (Edmucin) or the pericyte marker NG2. Note that CCN1:GFP-positive cells are endomucin-negative and that the CCN1:GFP signal localized predominantly in NG2-positive cells of neovascular tufts.

To determine whether the expression and tissue distribution of CCN1 is altered in response to ischemic insult, we used a mouse model of oxygen-induced retinopathy (OIR), which recapitulates key aspects of the neovascular response seen in humans with retinopathy of prematurity (ROP). In this model, exposure of mouse pups to hyperoxia (75% oxygen) for 5 days induced vasoobliteration of the central retina (Fig. 2A). Upon return to room air, the vascular repair response led to the aberrant formation of neovascular tufts growing towards the vitreous (Fig. 2B), a hallmark of ischemic retinopathies in humans^20^. In this model, the exposure to hyperoxia reduced the steady state mRNA levels of CCN1 by 72% (Fig. 2C). CCN1 gene expression was still downregulated during the subsequent hypoxic/ischemic phase by 50% in comparison to its levels under normoxic conditions (Fig. 2D). Exposure of the CCN1 promoter:GFP mouse line to OIR showed that the CCN1:GFP signal was localized pericellularly outside the endomucin-positive ECs in the vascular wall, the characteristic location for pericytes (Fig. 2E). GFP-positive pericytes, which have been characterized by their bipolar or multiprocessed morphology and wood knot-like vascular localization, ensheathe the endomucin-positive ECs^21^ lining the blood vessels. Consistent with this observation, CCN1:GFP and NG2-positive signals largely overlap on the preretinal neovascular tufts at P17 (Fig. 2F). Conceivably, pericyte-derived CCN1 could dramatically impact the pericyte secretome during the ischemic phase of OIR.

### Effects of pericyte-specific deletion of CCN1 on retinal angiogenesis

**Figure 3:**
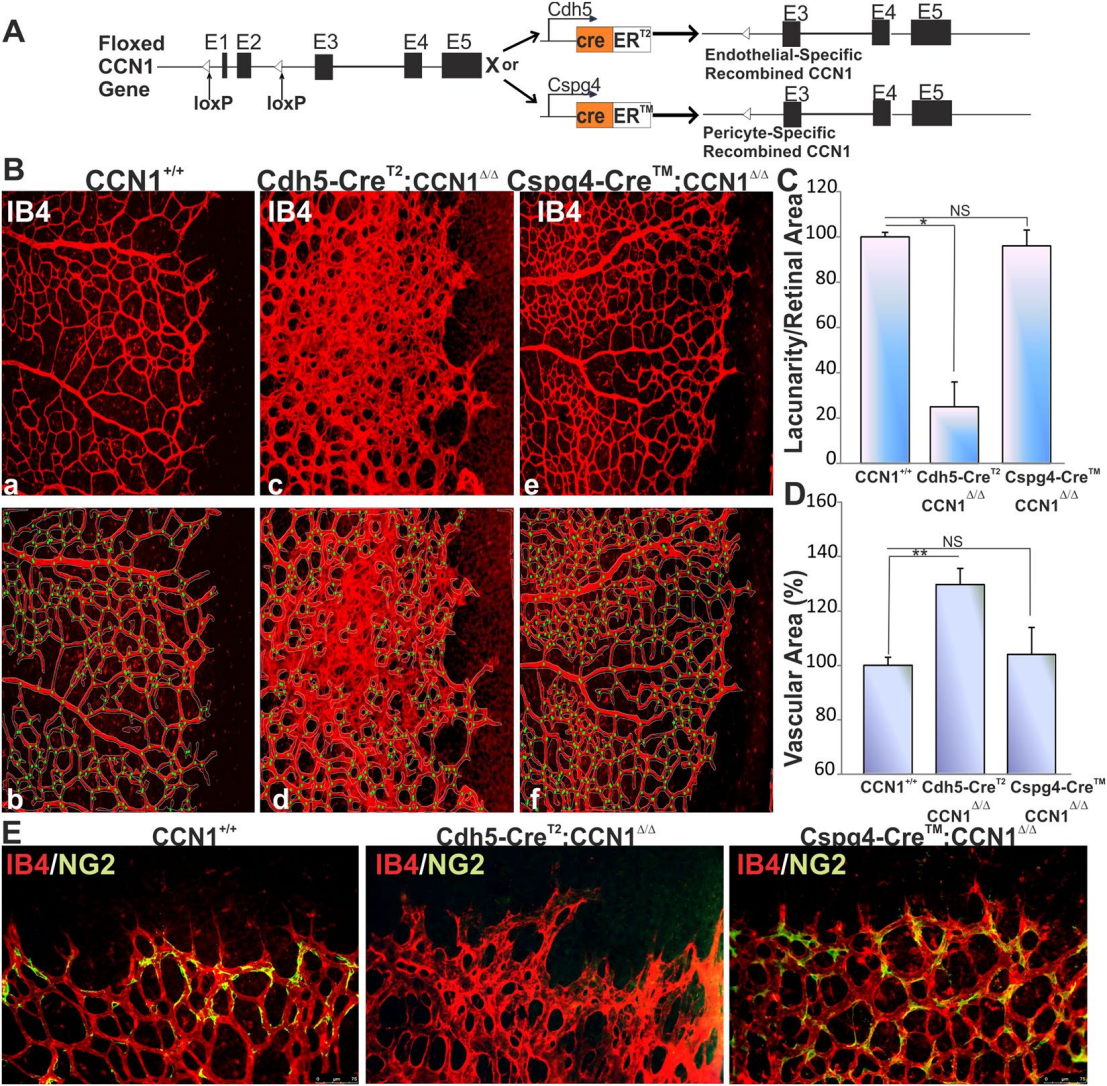
Effects of cell type-specific CCN1-deficiency on retinal vessel growth and development. (**A**) In the targeted CCN1 locus, exons 1 and 2 were flanked by loxP sites. Endothelial- or pericyte-specific promoter-mediated tamoxifen-induced cre excision removed the floxed fragment inactivating the CCN1 gene in the targeted cells. (**B**) Representative immunofluorescence images of IB4-stained whole retinal mounts of wild-type CCN1^+/+^ mice and their littermate with either endothelial- or pericyte-specific CCN1 deletion. Vascular parameters were analyzed using the AngioTool Software (b for a, d for c, f for e). The outline of the vasculature is shown in white. The vasculature skeleton representation is shown in red and branching points in green. (**C**,**D**) Quantitative analysis of vascular parameters of representative retinas of CCN1^+/+^ and CCN1 mutant mice. Graphical representations of the changes in vascular surface and lacunarity (i.e., the degree of “gappiness”) are shown. *p < 0.001 and **p < 0.05 (n = 5). (**E**) Representative immunofluorescence images of dual IB4 (red) and NG2 (green) staining of whole mount retinas of wild-type CCN1^+/+^ mice and their littermate with either endothelial- or pericyte-specific CCN1 deletion.

To investigate the cell-autonomous role of CCN1 during vascular development and pathology, we combined the CCN1 flox allele with either Cdh5-CreER^T2^ or Cspg4-CreER^TM^ allele for endothelial- and pericyte-specific deletion of CCN1 respectively (Fig. 3A). Tamoxifen-induced Cre recombinase expression under the control of the Cdh5 promoter resulted in severe vascular defects including enlargement of blood vessels and reduced lacunarity among vascular branches leading to the formation of vascular networks lacking the normal hierarchical arrangement of blood vessels (Fig. 3B–D), which is consistent with previous reports^18^. In this model, EC hyperproliferation within the capillary wall caused an excessive vascular enlargement, crowding of EC bodies and destabilization of junctional complexes that are likely to increase both sensitivity to shear stress forces by blood flow and leakiness. Conversely, tamoxifen-induced recombination in Cspg4-positive cells resulted in a seemingly normal wild-type-like vascular phenotype including normal vessel density, specification of blood vessels into arteries, capillaries and veins and adequate pericyte coverage (Fig. 3E).

### Pericyte-derived CCN1 exacerbates neovascular growth following ischemia

**Figure 4:**
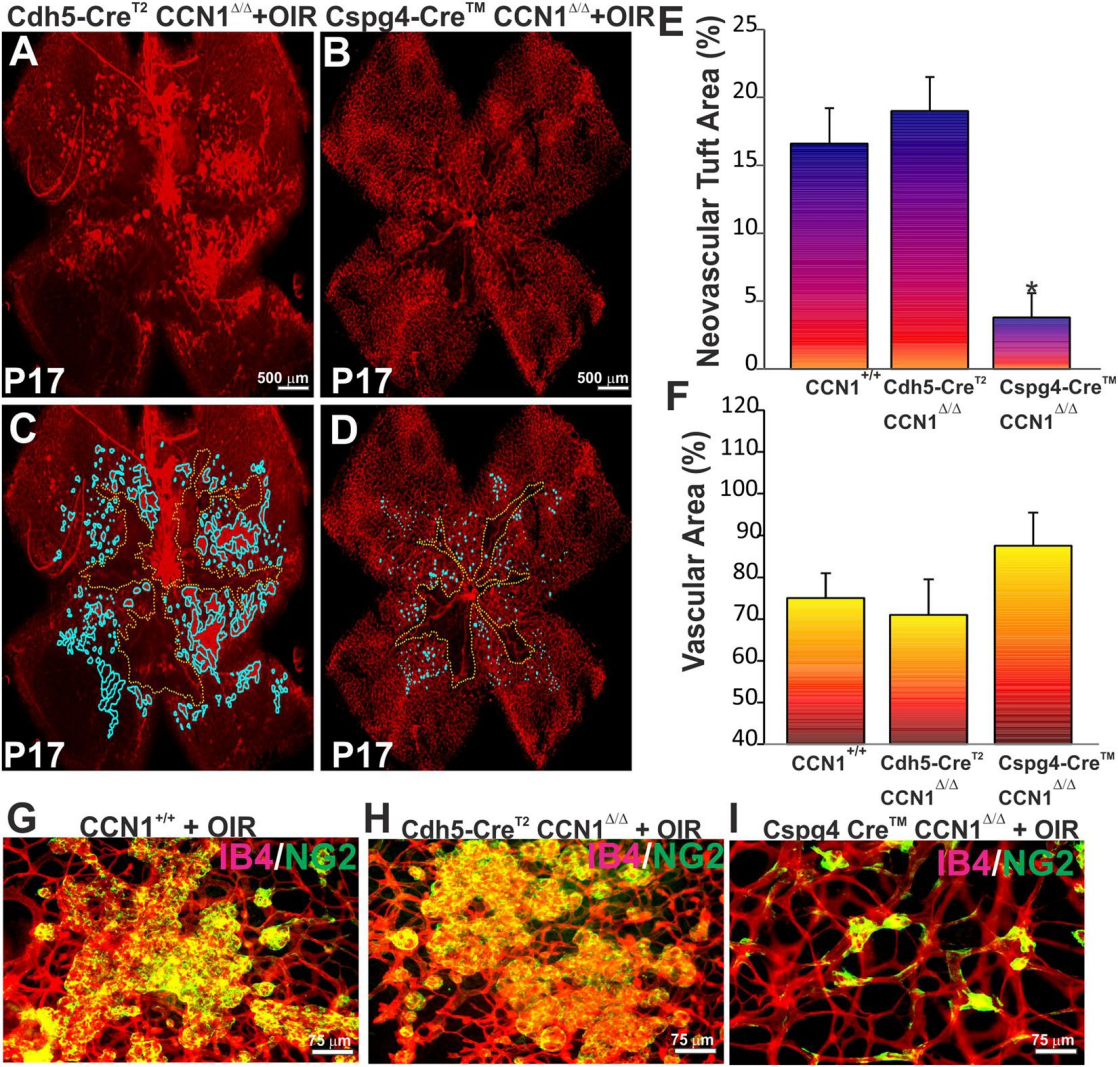
Effects of pericyte-derived CCN1 on pre-retinal neovascularization following OIR. (**A**,**B**) Representative flat-mount preparations of IB4-stained retinas from CCN1^+/+^, Cdh5-CreER^T2^ CCN1^Δ/Δ^ and Cspg4-CreER^TM^ CCN1^Δ/Δ^ mice following OIR. (**C**,**D**) Areas of pre-retinal neovascular tufts and vaso-obliteration were determined by computer-assisted image analyses (C for A and D for B). (**E**,**F**) Compiled data showing percentage of neovascular tuft and vascularized areas in Cdh5-CreER^T2^ CCN1^Δ/Δ^ and Cspg4-CreER^TM^ CCN1^Δ/Δ^ mouse retinas. Levels of neovascular and vascularized areas were set to 100% in wild-type OIR mice to facilitate comparisons among animals and groups. *p < 0.001 *versus* CCN1^+/+^ and Cdh5-CreER^T2^ CCN1^Δ/Δ^ (*n* = *4*). (**G**–**I**) Double IB4 and NG2 staining of neovascular tufts in retinas from CCN1^+/+^, Cdh5-CreER^T2^ CCN1^Δ/Δ^ and Cspg4-CreER^TM^ CCN1^Δ/Δ^ mice depicting neovascular tufts in central retinal areas following OIR. Note the reduced size of tufts (i.e., “microtufts”) in Cspg4- CreER^TM^ CCN1^Δ/Δ^ mice.

To determine whether pericyte-derived CCN1 contributes to neovascular growth under ischemic conditions, we subjected mice with pericyte-specific CCN1 deletion to OIR. Upon exit from the hyperoxic phase at P12, Cdh5-Cre CCN1^Δ/Δ^ and Cspg4-Cre CCN1^Δ/Δ^ mice exhibited a similar retinal vaso-obliteration pattern (data not shown). As expected, mice with EC-specific loss of CCN1 exhibited an extensive amount of neovascular tufts in the central retina (Fig. 4A). Conversely, mice with pericyte-specific loss of CCN1 function showed a markedly reduced neovascularization of the retina (Fig. 4B). Loss of CCN1 function in pericytes resulted in 78% and 82% reduction of aberrant neovascularization when compared with CCN1^+/+^ and Cdh5-Cre CCN1^Δ/Δ^ mice respectively (Fig. 4C–E). There was a simultaneous trend toward retinal revascularization following loss of pericyte-derived CCN1 (Fig. 4F) although the increase was not statistically significant. Of note, CCN1 deletion in pericytes resulted in the appearance of smaller tufts or “microtufts” composed largely of a few ECs capped with NG2-positive pericytes on their abluminal surface (Fig. 4G–I). Therefore, pericyte-specific loss of CCN1 reduces angiogenic signals that promote neovascular growth in OIR mice.

### Differential regulation of Wnt signaling by CCN1 in ECs and pericytes

**Figure 5:**
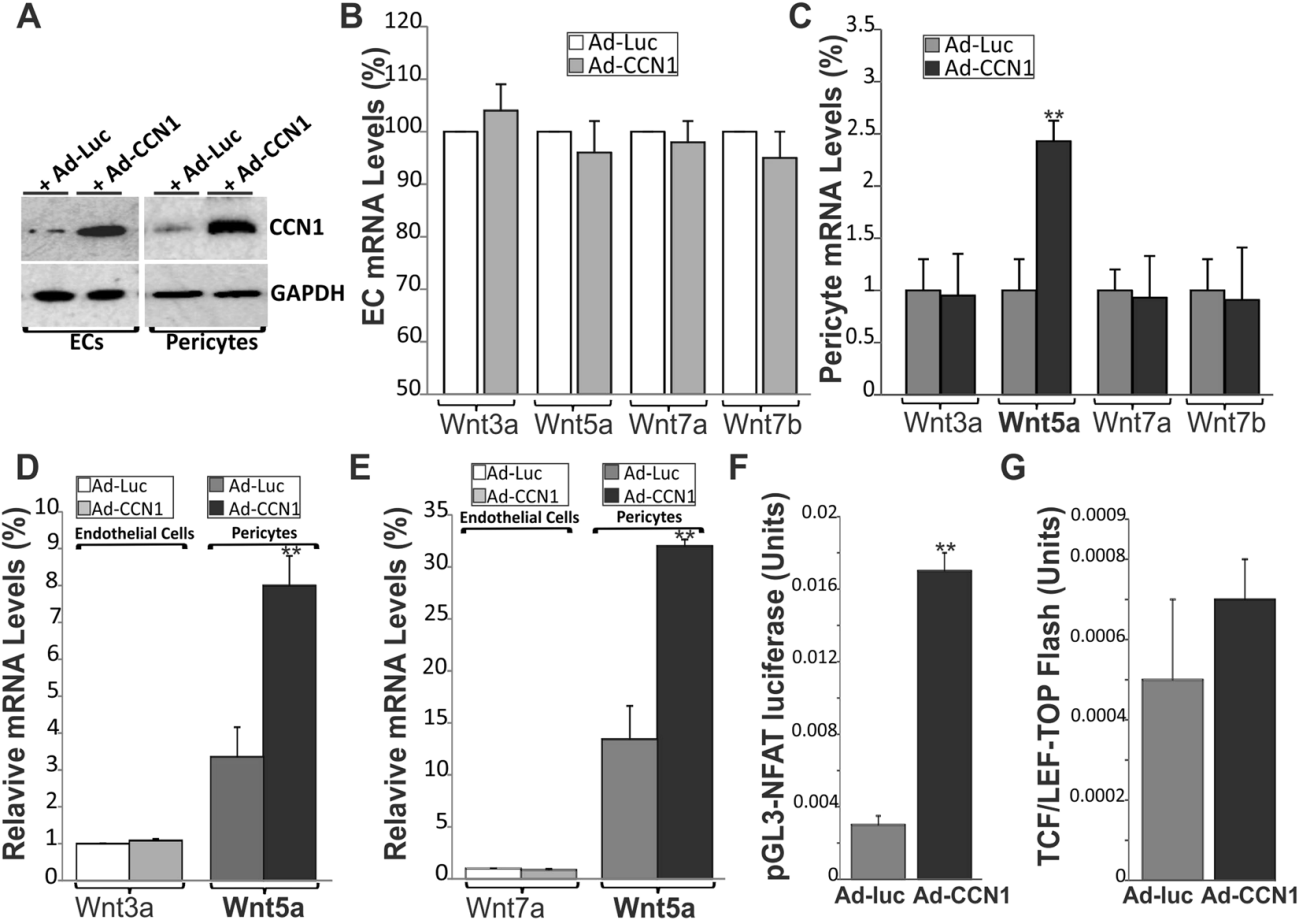
Adenovirus-mediated expression of CCN1 induced Wnt5a expression and activity in cultured pericytes. (**A**) Cultured ECs and pericytes were transduced with an adenoviral vector expressing either luciferase (Ad-luc) or CCN1 (Ad-CCN1). After incubation in serum-free medium for 16 h, cell lysates were analyzed by western immunoblotting for CCN1 protein expression. (**B**,**C**) Expression Wnt ligands was analyzed by qPCR in Ad-luc- and Ad-CCN1-infected cells. *p < 0.001 *versus* Ad-luc. (**D**,**E**) The relative abundance of Wnt5a transcripts and Wnt3a and Wnt7a was calculated by comparing their ΔCTs in pericytes *versus* ECs. (**F**,**G**) Effects of CCN1 on canonical and non-canonical Wnt signaling activity. Following adenoviral-mediated gene transfer with either Ad-CCN1 or Ad-luc, cultured pericytes were transiently transfected with either pGL3-NFAT luciferase reporter or LEF/TCF-M50 Super 8× TOP Flash plasmid. Luciferase activity was determined in cell lysates and media. Values shown are from a representative experiment performed in triplicate. **p < 0.01 *versus* Ad-luc. Experiments were repeated three times using different cell preparations with similar results.

On the basis of previous studies showing functional interactions between CCN1 and the Wnt signaling pathway^22, 23^, we examined the effects of CCN1 on the expression pattern of several Wnt ligands in cultured ECs and pericytes. Adenovirus-mediated overexpression of CCN1 in cultured ECs and pericytes increased CCN1 protein expression as determined by western immunoblotting (Fig. 5A). Under these conditions, CCN1 did not alter the endogenous expression of Wnt3a, Wnt7a, Wnt7b and Wnt 5a, most of which were weakly expressed in non-stimulated and CCN1-stimulated ECs (Fig. 5B). In contrast, overexpression of CCN1 in pericytes induced a 2.5-fold increase of Wnt5a but had no effect on the expression of other Wnt ligands (Fig. 5C). Wnt5a was more abundantly expressed in CCN1-stimulated pericytes than any other Wnt family member. In addition, the amount of Wnt5a expressed by pericytes was eight and thirty two times higher than the amount of Wnt3a and Wnt7a produced by ECs respectively (Fig. 5D,E). Functionally, Wnt ligands bind to the Frizzled family of receptors and trigger intracellular responses through canonical and/or noncanonical pathways^24^. Wnt5a mediates non-canonical Wnt signalling through the activation of the Wnt/Ca^2+^ via nuclear factor of activated T-cells (NFAT) or the Wnt/planar cell polarity pathway through Wnt co-receptors such as Kny, Ror2 and Ryk. Transfection experiments showed that the activity of a non-canonical NFAT-luciferase reporter was significantly increased in Ad-CCN1-infected pericytes compared with those overexpressing the Ad-luc vector control (Fig. 5F). Activity of the canonical TCF/Lef reporter was not significantly altered upon overexpression of CCN1 in pericytes suggesting that Wnt5a is a specific target of CCN1 signaling in pericytes (Fig. 5G).

### Functional interaction between CCN1 and Wnt5a in ECs

**Figure 6:**
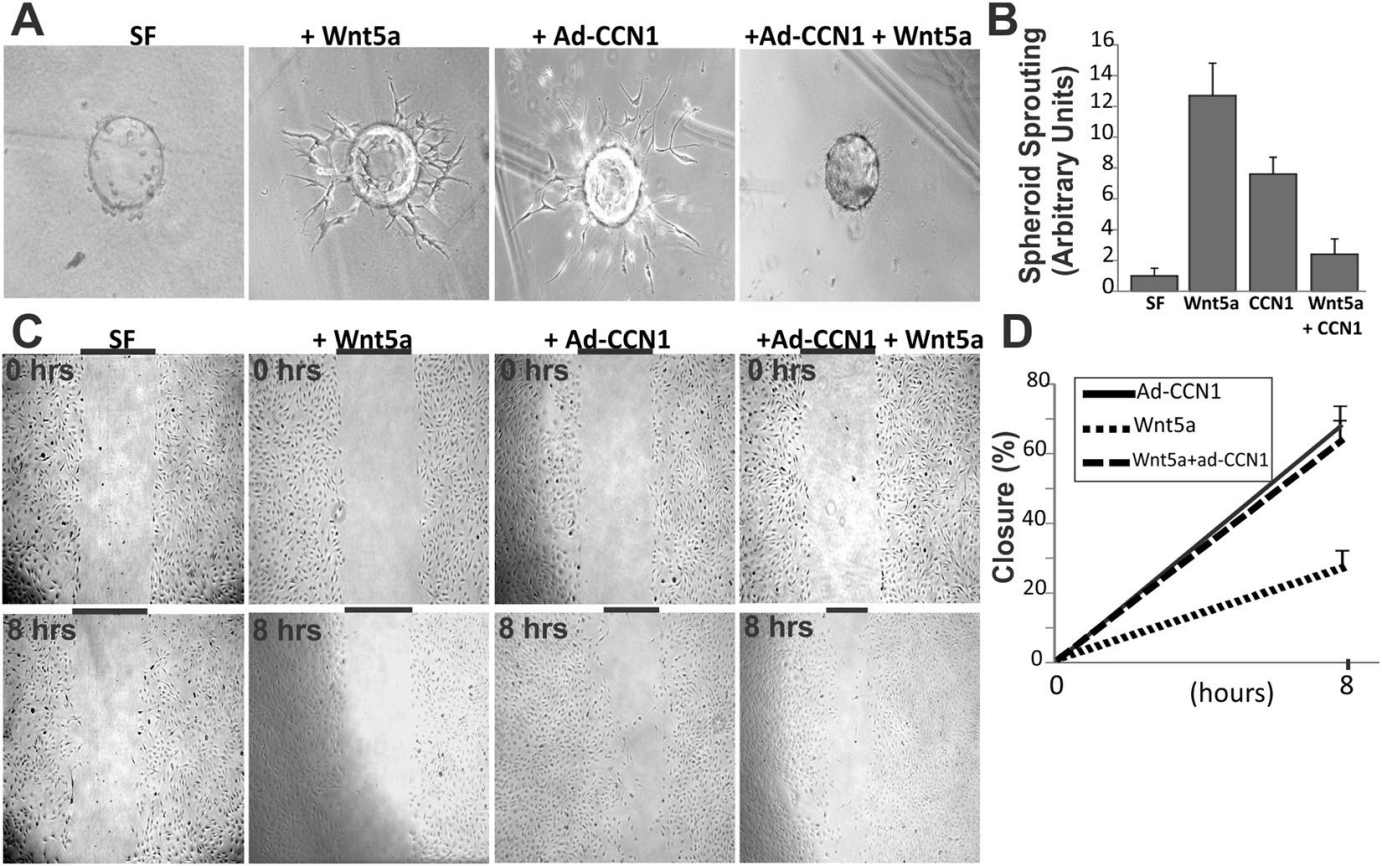
Functional and regulatory relationship between CCN1 and Wnt5a in ECs. (**A**,**B**) ECs were transduced with Ad-CCN1 or Ad-luc adenoviral vector, allowed to adhere to cytodex beads and then embedded in fibrin gels in the absence and presence of Wnt5a. Sprout formation was monitored by microscopy after 3 days. Quantitative analyses of spheroid sprouting was determined and expressed in arbitrary units. (**C**,**D**) ECs were pretreated with 5-FU to inhibit cell proliferation and the effect on cell migration was determined with cell scratch test. A representative experiment of the percentage closure from cell front after 8 h is shown.

Since Wnt5a is a major pericyte-derived paracrine signal that potentially regulates EC behavior *in vivo*, we further examined the effects of Wnt5a on EC growth and the compatibility of Wnt5a with CCN1 signals during EC sprouting. Using an *in vitro* spheroid sprouting assay which closely models vascular sprouting *in vivo*, we found that Wnt5a stimulated EC sprouting in a fibrin gel (Fig. 6A,B). ECs sprouted out of the beads and formed tubular networks within 3 days. Conversely, when the beads were coated with ECs overexpressing CCN1, cells migrated individually from the coated beads but they did not seem to recapitulate the sprouting process entirely including cell alignment and tube formation. Similarly, overexpression of CCN1 in ECs interfered with Wnt5a-induced EC sprouting suggesting that CCN1 signals override those of Wnt5a. In a two-dimensional model of EC wound, CCN1 overexpression alone or in the presence of Wnt5a increased migration of ECs and closure of wounded areas by 65 and 68% respectively (Fig. 6C,D). However, Wnt5a alone did not induce wound closure suggesting that Wnt5a had no effect on EC migration. Together, these data show that while CCN1-derived signals promote cell migration, they also interfere with Wnt5a-induced EC proliferation.

### Inhibition of Wnt5a suppresses pathological retinal neovascularization

**Figure 7:**
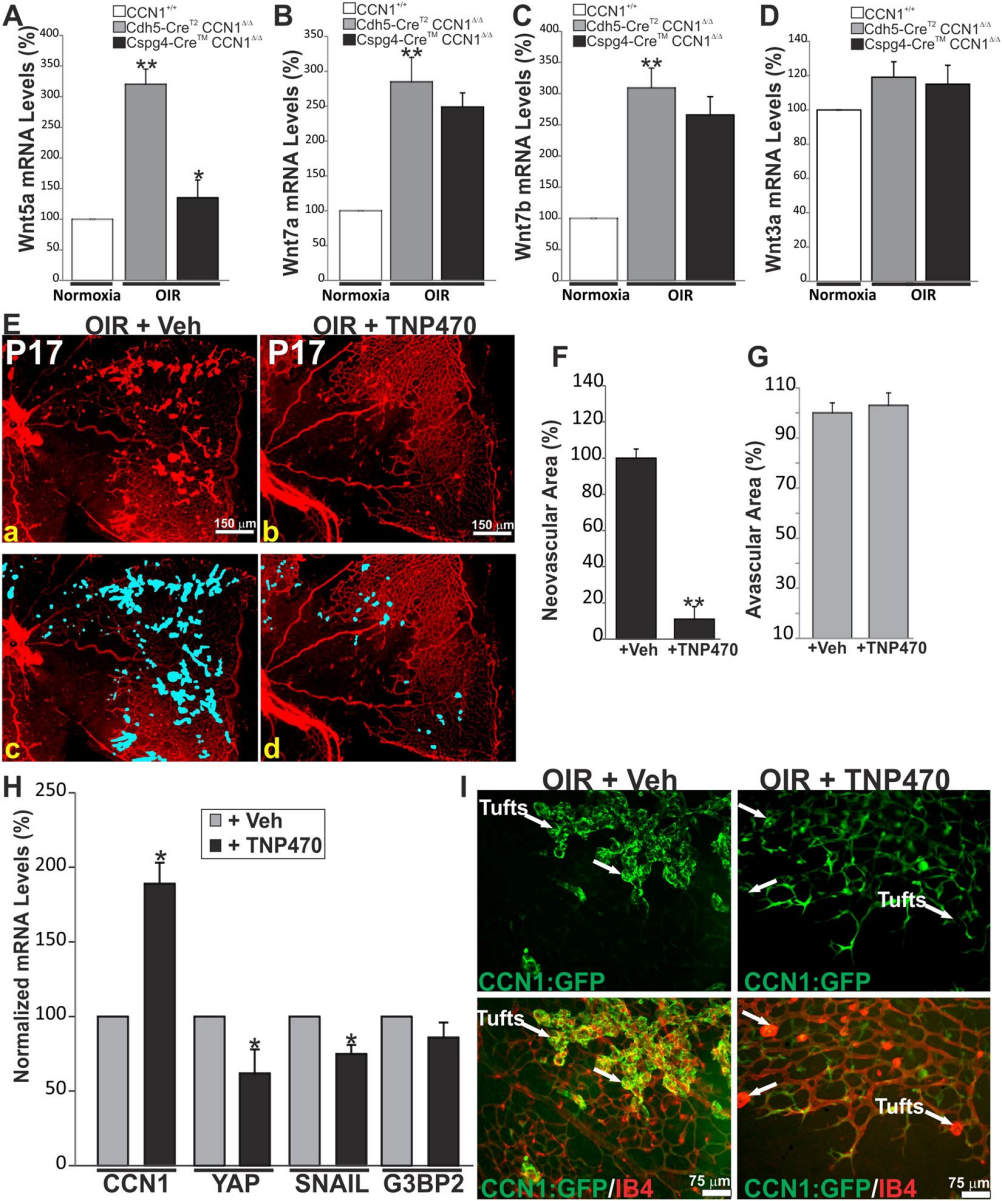
Inhibition of non-canonical Wnt5a reduced pathological neovascularization following OIR. (**A**–**D**) Expression of Wnt5a, Wnt7a, Wnt7b and Wnt3a in mice with either endothelial- or pericyte-specific CCN1 deletion and their wild-type littermates following OIR. Wnt ligand gene expression was analyzed by qPCR in retinal lysates harvested at P17. **p < 0.01 *versus* CCN1^+/+^. *p < 0.01 *versus* Cdh5-CreER^T2^ CCN1^Δ/Δ^. (**E–G**) Flat-mount images of IB4-stained retinas from OIR mice at P17 following treatment with either TNP470 or vehicle alone (Veh). Areas of pre-retinal neovascular tufts at P17 were delineated by a computer-assisted image software and highlighted in blue (c for a and d for b). The compiled data showing the percentage of neovascular and avascular areas are represented in F and G. **p < 0.05 *versus* +Veh. (**H**) Transcript levels of CCN1, YAP, SNAIL and G3BP2 as determined by qPCR in retinas from OIR mice injected with either control vehicle or TNP470. RNA levels at P17 (+Veh) were set to 100% to facilitate comparison among groups. *p < 0.01 *versus* P17 (+Veh). (**I**) Retinal flat-mounts from OIR CCN1:GFP reporter mice treated with vehicle or TNP470. Note that TNP470 treatment resulted in reduced neovascular tuft formation and reexpression of the CCN1:GFP reporter in ECs instead of perivascular pericytes in remnant tufts (arrows).

In wild-type mice, the expression of Wnt5a, Wnt7a, and Wnt7b was significantly increased in P17 mouse retinas following OIR whereas Wnt3a expression remained unchanged (Fig. 7A–D). Conversely, deletion of CCN1 in pericytes significantly reduced Wnt5a levels following OIR but it did not significantly alter those of Wnt7a, Wnt7b and Wnt3a suggesting that Wnt5a was specifically targeted by CCN1 under ischemic conditions. Depletion of pericyte-derived CCN1 reduced both Wnt5a and neovascular outgrowth following OIR. We further determined the pathophysiological significance of Wnt5a expression during OIR through injection of TNP470, a potent non-canonical Wnt inhibitor^25^. TNP470 was injected into the vitreous of P14 mouse pups following hyperoxic injury. Subsequent effects on retinal neovascularization were determined at P17. As shown in Fig. 7E,F, TNP470 significantly reduced pathological neovascularization by more than 80% in comparison to the vehicle alone. However, treatment with TNP470 did not promote a simulatneous revascularization of the retina (Fig. 7G). At he molecular level, TNP470 injection inhibited the expression of several Wnt5a target genes^26^ such as SNAI1 (Snail), Ras-GTPase activating protein SH3 domain-binding protein 2 (G3BP2), and Yes-associated protein (YAP) but upregulated the expression of the CCN1 gene (Fig. 7H). Concordantly, administration of TNP470 to the CCN1 promoter:GFP mouse line, shifted the CCN1:GFP signal from the pericyte-associated neovascular tufts to ECs lining the vascular wall (Fig. 7I). Taken together, these data support a major role of Wnt5a in neovascular growth under ischemic conditions.

### Effects of pericyte-derived Wnt5a on CCN1 expression and angiogenic behavior in ECs

**Figure 8:**
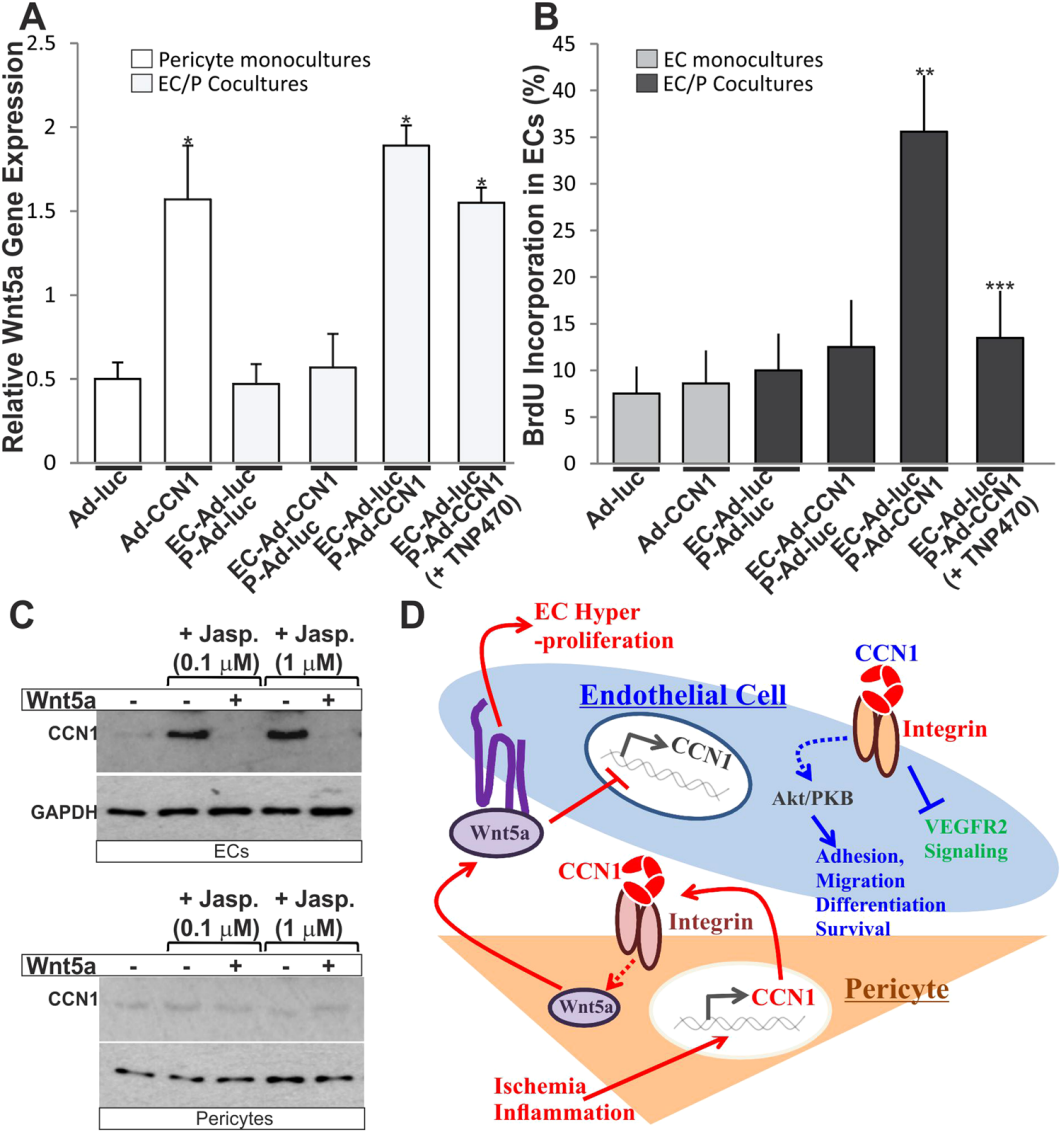
Regulation of EC proliferation and CCN1 gene expression by Wnt5a. (**A**,**B**) Monocultures and cocultures of ECs and pericytes (P) were transduced with either Ad-CCN1 or Ad-luc and further assessed for Wnt5a gene expression and BrdU incorporation in ECs. Cocultures of Ad-luc-infected ECs and Ad-CCN1-infected pericytes were incubated in the presence and absence of TNP470 (50 μM). *p < 0.01 *versus* Ad-luc. **p < 0.05 *versus* EC-Ad-CCN1 P-Ad-luc. ***p < 0.05 *versus* EC-Ad-luc P-Ad-CCN1. (**C**) Effects of Wnt5a on CCN1 gene expression on monolayer cell cultures of ECs and pericytes. Cultured ECs and pericytes were left untreated or treated with jasplakinolide for 2 h in the presence and absence of Wnt5a. CCN1 protein expression was determined by western immunoblotting. (**D**) Working model of the interplay between CCN1 and Wnt5a and their role in EC/pericyte crosstalk. Under ischemic conditions, EC/pericyte cooperation is altered by the shutdown of CCN1 in ECs and its expression by pericytes. Inasmuch as secreted CCN1 remains pericellular and acts largely on the cells that produce it, pericyte-derived CCN1 induces the expression of Wnt5a which acts in a paracrine manner to dampen CCN1 expression in ECs, stimulate their proliferation and promote retinal neovascularization. Thus, pericyte-derived CCN1 increases the sensitivity of ECs to ischemia.

The crosstalk between CCN1 and Wnt5a was further examined in EC-pericyte cocultures. In close agreement with the findings in OIR retinas, transduction of pericytes with Ad-CCN1 was associated with a significant up-regulation of Wnt5a expression in pericyte monocultures and a simultaneous increase of BrdU incorporation in ECs cocultured with pericytes (Fig. 8A,B). In the presence of TNP470, BrdU incorporation in ECs was significantly reduced suggesting a causal relationship between paracrine Wnt5a signaling and EC proliferation. Of note, CCN1 overexpression had no effect on EC proliferation. Thus, Wnt5a signaling evoked by pericytes was instrumental for EC hyperproliferation observed in the OIR model.

As an immediate-early gene encoded protein, CCN1 is rapidly induced in cell cultures in response to various stimuli primarily via actin-polymerization signals^27, 28^. However, CCN1 gene transcription was not induced after EC exposure to Wnt5a (data not shown). To determine whether Wnt5a potentiates or suppresses CCN1 gene induction in ECs, we examined CCN1 expression in EC mococultures exposed to jasplakinolide, a naturally occurring cyclic peptide that rapidly induces actin polymerization^29^. As shown in Fig. 8C, incubation of ECs with jasplakinolide strongly increased CCN1 protein levels. However, co-incubation with Wnt5a completely suppressed jasplakinolide-induced CCN1 gene expression. Thus, Wnt5a is a potent suppressor of CCN1 gene expression in ECs. Pericyte incubation with jasplakinolide did not increase CCN1 levels suggesting that CCN1 gene expression in pericytes and ECs is regulated through distinct mechanisms. Thus, pericyte-derived Wnt5a suppresses CCN1 gene expression in ECs in agreement with the finding that inhibition of Wnt5a signaling reestablished CCN1 expression in ECs in the model of OIR.

## Discussion

The interaction between ECs and pericytes through direct physical contacts and/or paracrine signaling molecules is an important mode of communication that is critical for vessel homeostasis and remodeling. Our study provided new insights into the mechanisms whereby pericyte-EC crosstalk partakes in pathological angiogenesis. We demonstrated that, under ischemic conditions, pericyte-derived paracrine signals modulate the neovascular growth response in the retina via the non-canonical Wnt ligand, Wnt5a. We provided evidence of a functional and regulatory relationship between the matricellular protein CCN1 and Wnt5a and showed how their dysregulation in ECs and pericytes contribute to the pathogenesis of ischemic retinopathy.

Our data showed that CCN1 was robustly expressed in angiogenic ECs during retinal vascular development and that only a residual CCN1 expression persisted in the mature vasculature. Pericytes may minimally, if at all, produce CCN1 during vascular development. Concordantly, mice with CCN1-deficient pericytes were phenotypically unimpaired and presented wild-type-like retinal vascular features. Conversely, the ischemic conditions in the retina under OIR forced the shutdown of CCN1 in ECs and induced its expression in pericytes. Differential regulation of the CCN1 gene in ECs and pericytes *in vivo* was likely due to the combined actions of several stimuli. The CCN1 gene is responsive to a wide range of extracellular stimuli including growth, inflammatory and stress factors in a cell type-dependent manner^13, 30^. Hypoxia, one of the most important driving factors in the pathogenesis of proliferative retinopathy, was described as an important inducer of CCN1 gene expression through both hypoxia-inducible factor-1α-dependent and -independent mechanisms although those effects were largely described in non-vascular cells (e.g., cancer and transformed cells)^31, 32^. In addition, an intricate system of checks and balances controls CCN1 protein levels including (i) transcriptional regulation through transactivation of the CCN1 gene promoter by constitutively expressed transcription factors (e.g., serum response factor, MRTF-A, YAP/TEAD)^1, 33, 34^; (ii) posttranscriptional control by miRNA (e.g., miR-155)^35^ and (iii) translational modifications (e.g., glycosylation) and degradation by proteases (e.g., MMPs, kallikreins)^36, 37^. A consensus finding in the literature is that physiological stimuli affect CCN1 gene expression by altering cytoskeletal actin dynamics (i.e., the polymerization state of actin)^28, 38^. In the current study, the role of actin in the upregulation of the CCN1 gene is demonstrated by the use of jasplakinolide, an actin filament polymerizing and stabilizing drug. In pericytes, jasplakinolide had no effect on CCN1 gene expression whereas EC treatment with jasplakinolide resulted in a superinduction of the CCN1 gene. The role of the actin cytoskeleton in CCN1 gene transcription in ECs may simply reflect the importance of specific components of the actin cytoskeleton in relaying signals between the cytoplasm and the nucleus. In particular, when F-actin levels in the cells are reduced in the absence of Rho-induced actin polymerization, G-actin inhibits transcription factors either directly or by sequestering cofactors required for their activation which consequently represses gene transactivation^39, 40^. Reversibly, the ability of jasplakinolide to increase F-actin levels, releases transcription factors from G-actin inhibition which promotes their translocation into the nucleus and subsequent transactivation of gene targets including the CCN1 gene. A recent study demonstrated that increased F-actin levels in the cells decreases phosphorylation of the transcriptional co-activator YAP which translocates into the nucleus and activates the CCN1 gene through interaction with TEA domain family member^41^. This regulatory pathway has been shown to be active in ECs^42^ which is in support of our data. It is conceivable that the mechanisms of F-actin-dependent activation of the CCN1 gene is cell type-specific and involves different transcription factors and/or actin isoforms in ECs and pericytes. Further studies to map out the transcriptional requirements of the CCN1 gene in ECs and mural pericytes during vascular development and regeneration are warranted.

Under ischemic conditions, EC/pericyte cooperation was altered by the shutdown of CCN1 in ECs and its expression by pericytes leading to reduced revascularization of the central retina, altered directional angiogenesis of the superficial capillary plexus and neovascular growth out of the retinal surface. Under these conditions, CCN1-induced Wnt5a expression in pericytes increased endothelial sensitivity to ischemia and enhanced EC proliferation (Fig. 8D). ECs minimally express Wnt5a although this non-canonical Wnt signaling factor was reported to act as a permissive rather than an instructive cue^43^. Wnt5a is critical for embryonic development because Wnt5a deficiency was associated with vascular defects in neural and skeletal tissues and reduced cell proliferation in tissues outgrowing from the primary body axis^44^. Wnt5a gene inactivation in mice led to increased vessel regression, decreased vascular density and a mild decrease in radial vascular expansion^43^. In the absence of Wnt5a, ECs of developing blood vessels have a reduced capacity to orient against the direction of blood flow specifically in vascular capillaries suggesting that Wnt5a signaling is required for stabilization of developing vascular networks by reducing endothelial shear sensitivity. In the adult, overproduction of Wnt5a has been commonly associated with tumor growth such as human melanoma and breast, pancreatic and gastric cancer^45^. These effects parallel those observed in the ischemic retina as the excessive production of Wnt5a by pericytes contributed to EC hyperproliferation and neovascular tuft formation. At the molecular level, Wnt5a gene expression has been reported to be largely controlled at the transcriptional level. Refined integrative genomic analyses demonstrated that Wnt5a gene transcription is induced by inflammatory factors such as TNF-α, IL-1β and TGF-β through activation of a highly conserved NF-κB-binding site within the Wnt5a promoter^46^. Of note, inflammation plays an important role in the pathogenesis of neovascular growth in the retina^47^. CCN1 itself was reported to induce an NF-κB-dependent inflammatory gene program that may contribute to Wnt5a gene upregulation^48^. The transcriptional requirements for the Wnt5a gene in pericytes potentially define its responsiveness to specific CCN1-derived signals in these cells.

Another important outcome of our study design is that pharmacological inhibition of Wnt5a signaling through injection of TNP470 significantly diminished neovascular tuft formation, which further supports the hypothesis that pericyte-derived Wnt5a acts in a paracrine manner to exacerbate endothelial growth during OIR. TNP470 is a synthetic analog of fumagillin, which has been shown to inhibit tumor growth in several clinical trials^49^. TNP470 blocks non-canonical Wnt signaling by targeting methionine aminopeptidase-2, a cytoplasmic metalloenzyme responsible for removing the N-terminal methionine from nascent proteins^50^. Intravitreal injection of TNP470 reduced neovascular growth in OIR retinas but did not promote revascularization of the central retina. Consistent with this finding, a study by Korn *et al*. showed that postnatal treatment of mice with TNP470 reduced vessel area and impaired radial expansion in the retina which is consistent with the antiproliferative activity of TNP470^51^. Another study by Ingber *et al*. showed that TNP470 treatment was rescued by non-canonical constitutively active disheveled 2 (Dvl2) mutant construct which further supports Wnt5a signaling as a major target of TNP470^52^. Wnt5a interacts with various membrane receptors including Frizzled-2, Frizzled-5 and Frizzled-7, ROR1, ROR2 and RYK^53^. Through these interactions, Wnt5a transduces signals through DVL-RhoA-ROCK, DVL-RhoB-Rab4, DVL-Rac-JNK, DVL-aPKC, calcineurin-NFAT, MAP3K7-NLK, MAP3K7 and NF-kB signaling cascades in a cell type- and context-dependent manner^54^. Concordantly, the expression of typical Wnt5a target genes such as YAP, SNAIL and G3BP2 was significantly downregulated upon intravitreal injection of TNP470 to wild-type animals subjected to OIR. Importantly, Wnt5a inhibition induced, at least in part, endothelial-specific re-expression of CCN1, which is an important guidance cue for normal patterning of the retinal vasculature. This might have contributed to reduced neovascular tuft formation in OIR mice treated with TNP470 as well. These observations further argue in favor of a regulatory relationship and functional interaction between Wnt5a and CCN1. Thus, dysregulated expression of these factors under ischemic conditions creates an environment that is not conducive for controlled growth and normal vessel regeneration.

In summary, our study uncovered a novel CCN1/Wnt signaling regulatory axis with a major regulatory function in pathological angiogenesis. We reported for the first time a mutual regulatory relationship between Wnt5a and the matricellular protein CCN1 affecting the coordinated crosstalk between ECs and pericytes. Both cell types act as a functional and physical unit in which the flow of molecular signals from one cell to another determines the angiogenic outcome in the tissue. Pharmacological manipulation of the molecules targeting intercellular communication and/or routes between ECs and pericytes will be beneficial in harnessing aberrant growth of blood vessels in different pathologies.

## Methods

### Mice

All procedures involving experimental animals were performed in accordance with the NIH Guide for the Care and Use of Laboratory Animals and all animal experiments were approved by the Institutional Animal Care and Use Committee of SUNY Downstate Medical Center. Mice were kept in a 12-h light-dark cycle, had free access to food and water and were maintained in pathogen-free conditions. CCN1:GFP mice carrying GFP under the control of the CCN1 promoter were obtained from the Mutant Mouse Regional Resource Center^19, 55^. Initially generated in the FVB/N-Swiss Webster background, CCN1:GFP mice were backcrossed for >10 times in the C57BL/6J genetic background. CCN1^flox/flox^ mice carrying loxP-flanked sequences around exon 1 and 2, and Tg(Cdh5-Cre ER^T2^) and Tg(Cspg4-Cre ER^TM^) BAkik transgenic mouse lines expressing the Cre recombinase gene under the pericyte-specific NG2 and ubiquitin gene promoter respectively have been previously described^56–58^. CCN1^flox/flox^ mice were crossed with Cdh5-Cre ER^TM^ and Cspg4-Cre ER^TM^ mice to induce EC- and pericyte-specific deletion of CCN1 respectively. Genotyping was determined by qPCR to identify mice with floxed alleles and hemizygous floxed allele with and without the Cre allele. A solution of 4-hydroxytamoxifen (HT) was dissolved in ethanol at 10 mg/ml, and then 4 volumes of corn oil were added. HT samples were further diluted in corn oil prior to intraperitoneal injection of 100 μl to mouse pups. Recombination levels in mice with the Cre allele were determined by qPCR and western blot as described previously^59^. Mice with recombination efficiency >95% were processed for further analyses.

### OIR and determination of vascular parameters

Neonatal mice and their nursing dams were exposed to 75% oxygen in a PRO-OX 110 chamber oxygen controller (Biospherix Ltd, Redfield, NY) between P7 and P12 producing vaso-obliteration and cessation of vascular development in the capillary bed of the central retina. On P12, mice were placed in normoxic conditions for up to 5 days (P17) for maximum neovascular response. For controls, room air mice were raised under normal light and temperature conditions. Mice were euthanized at the indicated time periods as per our approved animal protocol. Eyes were enucleated and processed for immunohistochemical and molecular analyses as described in the text.

### Intravitreal injection of TNP470

TNP470 (50 μM) was injected intravitreally at p14 using a 33-gauge needle. In the contralateral eye, an equal volume of vehicle (dimethyl sulfoxide) alone was injected. Retinas were dissected and processed for molecular and immunohistochemical analyses as described in the text.

### Immunohistochemical staining and quantification of retinal vasculature

Retinas were dissected, laid flat on SuperFrost^®^ Plus coated slides and permeabilized in 0.1% Triton X-100 at room temperature for 20 min. Blood vessels were visualized by IB4 staining as previously described^18^. Immunostaining with primary antibodies was performed overnight at 4 °C. The following day, retinas were washed 2 times for 5 minutes each in phosphate buffer solution (PBS) and incubated with a secondary antibody overnight at 4 °C. Retinas were mounted in a Vectashield mounting medium (Vector Laboratories, Burlingame, CA) and images were captured using a Leica DM5000 B fluorescence microscope. Fields of view of the retinal vascular networks from control and mutant mice were captured by using the 40x objective lens and included regions of capillary-sized vessels directly adjacent to radial arterioles. Vascular parameters were measured using the AngioTool software^60^. For each parameter, at least 4 fluorescent images/retina were taken. In OIR mice, the areas of vasoobliteration were measured by delineating the avascular zone in the central retina and calculating the total area using Photoshop CS5 (Adobe). Similarly, the areas of pre-retinal neovascularization (i.e., neovascular tufts) were calculated by selecting regions containing tufts, which appear more brightly stained than the normal vasculature, based on pixel intensities. Selected regions were then summed to generate a total area of neovascularization. The avascular and neovascular areas were expressed as a percentage of the total retinal area.

### Cell culture, adenoviral infection and transient transfection with plasmid vectors

Retinal ECs were obtained from Cellpro (San Pedro, CA) and pericytes were from obtained from QBM Cell Science Laboratories (Ottawa, Canada). Cells were maintained in culture according to the manufacturer’s instructions. EC phenotype was validated using several criteria: (i) “cobblestone” morphology appearance, (ii) positive staining for Factors VIII, (iii) uptake of acetylated low-density lipoprotein, and (iv) CD-31 and IB4 positivity. Pericyte phenotype was determined by immunostaining with NG2 and desmin antibodies. Cells were propagated in 35-mm dishes in predefined growth medium containing 10% of fetal bovine serum (FBS).

For cell infection with adenoviral vectors, ECs and pericytes at 80% confluency were first incubated in serum-free medium for up to 3 h with adenoviral vectors expressing either CCN1 (Ad-CCN1) or luciferase (Ad-luc). Cells were further incubated in serum-containing medium for 16 h. All adenoviruses were replication-deficient and used at 20 multiplicities of infection. Production of adenoviral vectors was performed in the Preclinical Vector Core Laboratory at the University of Pennsylvania under the auspice of The Gene Therapy Resource Program of the NHLBI of the National Institutes of Health.

Transfection with plasmids was performed using FuGENE 6 transfection reagent in serum-free medium according to the manufacturer’s specifications (Roche Diagnostics). Cells were transfected with expression vectors 8 × Tcf/Lef promoter-luciferase reporter and 4 × NEF promoter-reporter luciferase and co-transfected with pRL-SV40 vector containing the *Renilla* luciferase gene to adjust for transfection efficiency. The FuGENE 6/DNA mixture was left on cells for 3 h. Cells were allowed to recover in fresh medium containing 10% serum. Cells were incubated in serum-free medium and cell lysates were prepared and assayed for luciferase activity levels. Firefly luciferase activity was normalized to that of Renilla luciferase.

### Fibrin-bead-based angiogenesis and migration assays

Cultured ECs were mixed with dextran-coated Cytodex 3 microbeads and incubated overnight in FBS-containing medium. Cell-coated beads were re-suspended in fibrinogen, aprotinin and thrombin and allowed to clot in 24-well tissue culture plates. A fibroblast feeder layer was added on top of the fibrin gel. Medium was changed regularly with new addition of the indicated reagent. EC sprouting was monitored for 3 days and scored for filopodia, angiogenic sprouts and dissociated ECs.

For endothelial migration experiments, cells were seeded at sub-confluence on day 1. On day 2, fluorouracil (5-FU) was added to suppress cell proliferation and a wound was made to the confluent cell monolayer. Migration of cells into the wounded area was determined 8 h later.

### EC-pericyte cocultures

ECs and pericytes were cocultured using culture plates and inserts (Transwells; Corning, Inc. NY) coated on their upper side with a solution of rat tail collagen (1 mg/ml). Before performing cocultures, EC and pericyte monocultures were transduced with either Ad-CCN1 or Ad-luc as described in the text. EC cultures were seeded into the plates while pericytes were plated on the Transwell inserts in a 1:1 ratio and incubated overnight in culture medium containing 10% FBS. Pericytes on inserts in 2 ml medium were then placed onto the plates containing ECs on the bottom in 1 ml culture medium. Alternatively, ECs were seeded in a transwell culture insert separated from the pericytes seeded in the well below. After a coincubation for 12 h in EC/pericyte medium (2% FBS) in the presence and absence of TNP470 (50 μM), cells on the plate and in the transwell were washed three times with ice-cold PBS. For EC proliferation, 1 μM BrdU (Sigma-Aldrich) was added to the culture medium for the duration of the colcultures. Cells were then incubated with monoclonal antibodies against BrdU, followed by fluorescein-conjugated secondary antibodies. BrdU-positive nuclei were counted in 4 fields of view. For molecular analyses, cells were processed as described below.

### RNA isolation and quantitative analysis of mRNAs

Total RNA was extracted from either cells or tissues using RNAEasy column purification protocol (Qiagen) according to the manufacturer’s instructions. Steady state levels of specific mRNAs were determined by qPCR using TaqMan technology on a StepOne ABI sequence detection system (Applied Biosystems, Carlsbad, CA). Highly specific primers were designed using Web-based primer design programs. Primers used included those for VEGF, CCN1, Wnt3a, Wnt5a, Wnt7a, Wnt7b, SNAIL, YAP and G3BP2. The cycling parameters for qPCR amplification reactions were: AmpliTaq activation at 95 °C for 10 min, denaturation at 95 °C for 15 s, and annealing/extension at 60 °C for 1 min (40 cycles). Triplicate *Ct* values were analyzed with Microsoft Excel using the comparative *Ct* (ΔΔ^Ct^) method as described by the manufacturer. The transcript amount (−2^ΔΔCt^) was obtained by normalizing to an endogenous reference (18S rRNA) relative to a calibrator.

### Antibodies and western immunoblotting

Cell and tissue lysates were homogenized in lysis buffer. Protein samples (20 μg) were fractioned in a 10% SDS-polyacrylamide gel, transferred to nitrocellulose membrane and western blot analysis was performed with each of the indicated primary antibodies. Immunodetection was performed using enhanced chemiluminescence (Pierce Biotech, Rockford, IL).

### Statistical analysis

Data were expressed as means ± S.E. To test differences among several means for significance, a one-way ANOVA with the Newman-Keuls multiple comparison test was used. Where appropriate, post hoc unpaired t-test was used to compare two means/groups, and p values <0.05 or <0.01 were considered significant. Statistical analyses were performed using the Prism software for Windows (GraphPad Inc, San Diego, CA).
